# Solitary fibrofolliculoma: a retrospective case series review over 18 years

**DOI:** 10.5935/0004-2749.20200092

**Published:** 2024-02-11

**Authors:** Cecilia Díez-Montero, Miguel Diego Alonso, Pilar I. Gonzalez Marquez, Silvana Artioli Schellini, Alicia Galindo-Ferreiro

**Affiliations:** 1 Department of Ophthalmology, Rio Hortega University Hospital, Valladolid, Spain; 2 Department of Pathology, Rio Hortega University Hospital, Valladolid, Spain; 3 Department of Ophthalmology, Faculdade de Medicina, Universidade Estadual Paulista “Júlio de Mesquita Filho”, Botucatu, SP, Brazil

**Keywords:** Birt-Hogg-Dubé, syndrome/pathology, Eyelid neoplasms, Skin neoplasms, Síndrome de Birt-Hogg-Dubé/patologia, Neoplasias palpebrais, Neoplasias cutâneas

## Abstract

**Purpose:**

The purpose of this study was to report a series of cases of solitary fibrofolliculoma, a lesion seldom observed in the lids. Demographics, as well as clinical and histological aspects of the lesion were evaluated.

**Methods:**

This was a retrospective case series spanning a period of 18 years. All the included patients were diagnosed with solitary fibrofolliculoma confirmed by histological examination. Data regarding patient demographics, signs, and symptoms, course of the disease, location of the lesion, clinical and histological diagnosis, and outcome were collected. **Result**s: Eleven cases of solitary fibrofolliculoma were diagnosed in the study period. The median age of patients was 51 ± 16.3 years (range: 27-78 years). Most patients were females (7/11; 64%). Five of the patients (45%) were asymptomatic; four (36%) reported bleeding, one (9%) had referred itching, and one (9%) rubbing of the lesion. The lesion occurred in a wide range of locations; one of them was located in the lids. The diagnosis for all lesions was histological based on characteristic findings of a hair follicle occasionally dilated and containing keratin material surrounded by a moderately well-circumscribed thick mantle of fibrous tissue. The infundibular follicular epithelium extended out into this fibrous mantle forming epithelial strands or cords. There were no relapses after exeresis.

**Conclusion:**

Solitary fibrofolliculoma is a rare lesion, seldom affecting the eyelids. We reported 11 cases, and the third case reported thus far in the literature affecting the lids. Diagnosis may be easily missed due to the nonspecific symptoms and clinical appearance. Therefore, it is necessary to perform excisional biopsy and histological examination for the recognition of this lesion.

## Abstract (pt)

**Objetivo:**

o objetivo deste estudo foi relatar uma série de casos de fibrofoliculoma solitário, uma lesão raramente observada nas pálpebras. Demografia, bem como aspectos clínicos e histológicos da lesão foram avaliados. **M**étodos: Trata-se de uma série de casos retrospectivos, com um período de 18 anos. Todos os pacientes incluídos foram diagnosticados com fibrofoliculoma solitário confirmado por exame histológico. Foram coletados dados referentes à demografia, sinais e sintomas dos pacientes, evolução da doença, localização da lesão, diagnóstico clínico e histológico e desfecho.

**Resultados:**

Onze casos de fibrofoliculoma solitário foram diagnosticados no período do estudo. A média de idade dos pacientes de 51 ± 16,3 anos (variação: 27-78 anos). A maioria dos pacientes era do sexo feminino (7/11, 64%). Cinco dos pacientes (45%) eram assintomáticos; quatro (36%) relataram sangramento, um (9%) referiu coceira e um (9%) fricção da lesão. A lesão ocorreu em luma ampla variedade de locais; um deles sendo nas pálpebras. O diagnóstico de todas as lesões foi histológico com base nos achados característicos de um folículo piloso ocasionalmente dilatado e contendo material de queratina, cercado por um manto espesso de tecido fibroso moderadamente bem circunscrito. O epitélio infundibular folicular se estendeu até esse manto fibroso, formando cordões ou cordões epiteliais. Não houve recaídas após exérese.

**Conclusão:**

Fibrofoliculoma solitário é uma lesão rara, mais ainda quando afeta as pálpebras. Relatamos 11 casos, e o terceiro relatado até o momento na literatura que afeta as pálpebras. O diagnóstico pode ser facilmente esquecido devido a sintomas inespecíficos e aparência clínica. Portanto, é necessário realizar biópsia excisional e exame histológico para o reconhecimento dessa lesão.

## INTRODUCTION

Fibrofolliculoma (FB) usually appears as a rare, small, clinically asymptomatic, skin-colored, dome-shaped lesion, typically located on the head, face, neck, and upper trunk^(1^-^3)^. Trichodiscoma, neurofollicular hamartoma, and spindle cell-predominant trichodiscoma are other designation related to FB in different stages of development of a single entity^(4^,^5)^. FB can arise as a solitary benign lesion or associated with multiple FB, termed Birt-Hogg-Dubé syndrome.

This benign tumor was first described by Birt et al. in 1977^(6)^. However, the first solitary periocular FB was reported in 2007^(7)^. Only 18 cases of solitary FB have been previously described (Table 1) and only two of those were located on the eyelids^(1^,^7)^.

**Table 1:** Previous literature reports (in English) of solitary fibrofolliculoma (FB)

Author-year (reference number)	Number of cases described	Location	Age (years)	Sex	Clinical diagnosis	Type of surgery	Recurrence
Scully et al., 1984^(14)^	1	Chin	62	Female	Intrademal nevus	Biopsy	Not specified
					Wart		
					Hemangioma Angiofibroma		
Gartmann, 1985^(15)^	1	Nose	85	Female	NA	NA	NA
Starink and Bownstein, 1987^(8)^	5	Chin (1) Nose (1)	46 (average)	Male Female Male Female Male	Epidermoid cyst (3) Fibroma (1)	Biopsy	Not specified
		Cheek (1)			Intradermal nevus (1)		
		Ear (1)					
		Eyebrow (1)					
Lee et al., 1996^(16)^	1	Chin	56	Female	NA	NA	NA
Hong et al., 1997^(17)^	1	Scalp	40	Female	Not specified	Excision biopsy	No
Pan and Sarma, 2006^(18)^	1	Nose	60	Male	Not specified	Excisional biopsy	Not specified
Park et al., 2007<^19)^	1	Ear	56	Male	NA	NA	NA
Chang et al., 2007^(7)^	1	Upper eyelid	37	Female	Chalazion	Pentagonal resection	No
Cesinaro et al., 2010^(20)^	1	Nose	63	Female	Basal cell carcinoma	Complete excision	No
Cho et al., 2012^(3)^	1	Ear	45	Male	Not specified	Shave biopsy	No
Bhattacharyya et al., 2015^(21)^	1	Upper eyelid	32	Male	Chalazion	Full thickness excision	No
Criscito et al., 2017^(2)^	1	Cheek	72	Female	Not specified	Not specified	Not specified
Riley et al., 2018^(22)^	1	Abdomen	54	Female	Not specified	Excisional biopsy	Not specified
Sohn et al., 2018^(11)^	1	Posterior auricular area	50	Male	Not specified	Shave biopsy	Not specified

NA= not applicable.

We reviewed our FB cases over a period of 18 years and presented the demographic, clinical, and histopathological characteristics of this rare condition. To the best of our knowledge, this is the largest series of cases involving solitary FB. Moreover, it included the third periocular FB case reported thus far in the literature.

## METHODS

This retrospective case series evaluated patients with solitary FB who underwent lesion exeresis with histological confirmation between 2000 and 2018 at the Rio Hortega University Hospital (Valladolid, Spain). The Institutional Research Board approved this study and consent was waived due to the retrospective nature of the study.

All consecutive cases that underwent lesion removal were included in this survey. The surgeries were performed by ophthalmologists, and plastic or maxillofacial surgeons; histopathology reports were dictated by specialist dermatopathologists. Data regarding demographic details (age and sex), course of the disease, location of the lesion, time of evolution preceding surgical treatment, clinical diagnosis and related systemic diseases, day of surgery, surgical technique, histopathologic diagnosis, and outcome (including recurrence) were collected. Data were analyzed according to the frequency of occurrence.

## RESULTS

**Figure 1:**
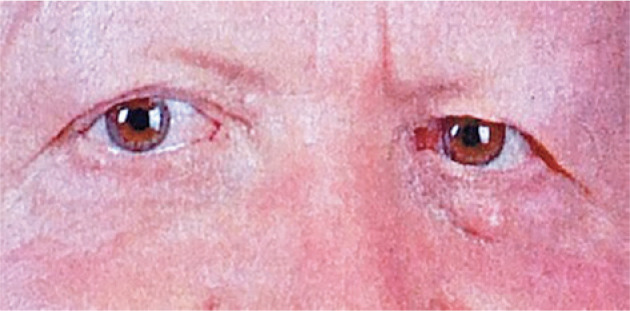
Solitary fibrofolliculoma (FB) flesh-colored, protruding mass observed on the left lower eyelid.

Eleven cases of solitary FB were observed during the study period. Table 2 presents the details of our case series. The median age of the patients (at the time of excision) was 51 ± 16.3 years (range: 27-78 years). Seven of those were females (64%) and four were males (36%). Five patients (45%) were asymptomatic, four (36%) reported bleeding, one (9%) had referred itching, and one (9%) reported rubbing of the lesion. The lesion appeared in a wide range of locations: three on the cheek (27%), three on the nose (27%), and one each on the lower eyelid, chin, ear, back, and forearm (9%, respectively). The lesion was solitary in seven patients (64%) and associated with other lesions in four (36%) patients: one patient (9%) who also presented with a seborrheic keratosis, one (9%) with a facial melanoma, one (9%) with dermatofibroma and hemangioma, and one (9%) with nevus, infundibular cyst, wart, and common skin lesions. Time to progression was, <6 months in two patients (18%), 6-12 months in one patient (9%), and >12 months in five patients (45%). Time to progression data of three patients (27%) were lost. The clinical diagnosis was “benign skin lesion” in five patients (45%), wart, fibroma, lipoma, and epidermal cyst in four patients (9%). The last two patients presented an unclear differential diagnosis (i.e., wart vs. fibroma and fibroma vs. nevus, respectively). In other words, the FB was not clinically recognized in any of the patients (Figure 1).

**Table 2:** Demographic and clinical characteristics of patients with fibrofolliculoma (FB) in Spain

Case no	Age	Sex	Time with lesion (months)	Symptoms	Clinical diagnosis	Location	Type of treatment	Follow-up after surgery (years) Recurrence		Related systemic disease
1	78	F	>12	None	Skin lesion	Nose	Shave biopsy	5	No	Facial melanoma
2	42	F	>12	None	Wart	Ear	Shave biopsy	NA	NA	None
3	59	F	>12	Bleeding	Skin lesion	Nose	Shave biopsy	NA	NA	None
4	27	F	NA	None	Skin lesion	Cheek	Shave biopsy	None	No	None
5	62	M	NA	Itching	Wart vs fibroma	Arm	Shave biopsy	None	No	None
6	39	F	<6	None	Skin lesion	Cheek	Excisional biopsy	3	No	Nevus, infundibular cyst, wart, skin lesions
7	39	F	NA	Brushing	Fibroma	Back	Shave biopsy	None	No	Dermatofibroma with hemangioma
8	62	M	>12	None	Epidermoid cyst	Chin	NA	None	No	None
9	37	F	6-12	Bleeding	Fibroma vs nevus	Nose	Shave biopsy	NA	NA	Nevus
10	44	M	>12	Bleeding	Skin lesion	Cheek	Excisional biopsy	None	No	None
11	72	M	<6	Bleeding	Lipoma	Lower eyelid	Excisional biopsy	1	No	None

F= female; M= male; NA= not applicable.

**Figure 2:**
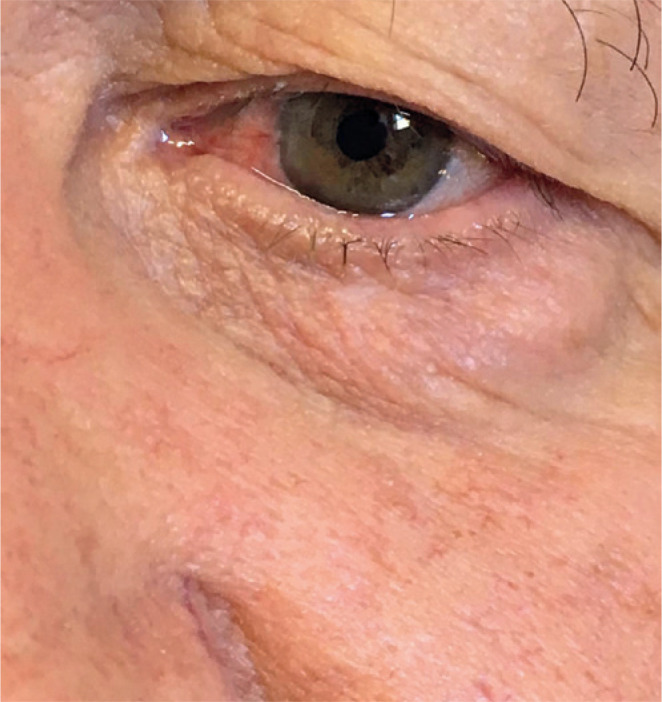
Postoperative examination at 1 year, revealing the absence of lesion recurrence.

**Figure 3:**
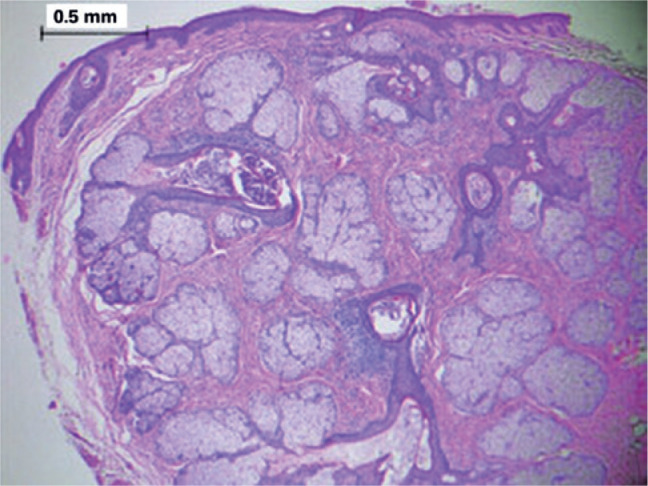
Low-power histopathologic appearance of a fibrofolliculoma showing proliferation of fibrotic stroma surrounding the central dilated infundibulum of the hair follicles with proliferative thin epithelial strands. (Hematoxylin-eosin stain: ×40).

**Figure 4:**
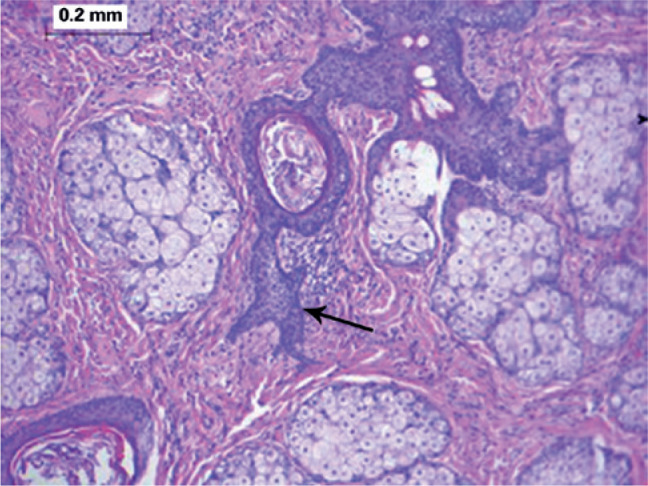
Characteristic high-power histopathologic image presenting the appearance of proliferating infundibular epithelial strands with pe rifollicular fibrous reaction. (Arrow: cords of basalioid cells; hematoxylin-eosin stain: ×100).

**Figure 5:**
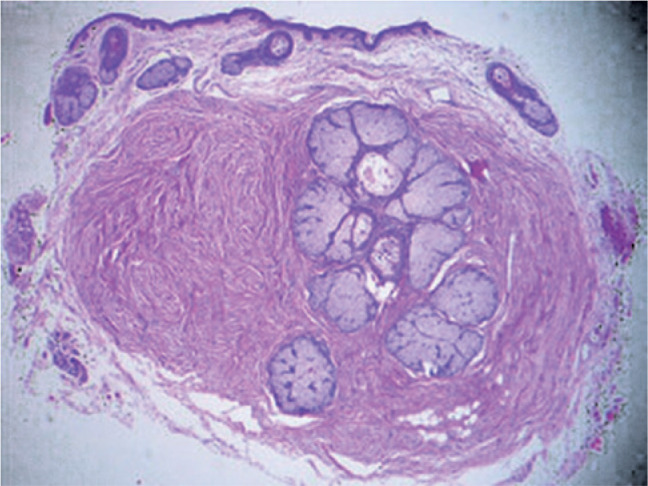
Features of fibrofolliculoma-trichodiscoma in the same lesion, shown through hematoxylin-eosin staining.

Histological examination revealed a well-defined tumor mass involving a group of adjacent pilosebaceous follicles and proliferative epithelial cords and spurs in the center, with a surrounding fibrous mesenchymal component. Characteristic proliferating infundibular epithelial strands with perifollicular fibrous reaction anastomosing to form an epithelial network were also observed (Figures 2-5).

Seven patients underwent a “shave” biopsy, whereas three patients had an excisional biopsy. Following surgery, all patients recovered well without any recurrence. Surgery data of one patient were lost.

No other lesions were detected on the face, neck, axillae, upper trunk, or groin. Patients did not report a family history of multiple skin papules or skin diseases. The final diagnosis for all the included lesions was solitary FB. None of the patients developed recurrence of the lesion after exeresis.

## DISCUSSION

To the best of our knowledge, this is the largest case series of solitary FB, a fibrotic hamartoma characterized by infundibular epithelial and perifollicular fibrous proliferation. During a period of 18 years, only 11 solitary FB cases were encountered at our center, resulting in a frequency of occurrence of 0.6 cases per year. According to the results of our review, only 18 cases of FB in its pure form and unassociated with other significant cutaneous findings had been published worldwide prior to our case series (Table 1). Moreover, the present case series is the second involving solitary FB preceded only by that reported by Starink and Brownstein^(8)^.

The median age of presentation in our solitary FB cases was 51 ± 16.3 years (range: 27-78 years), which is a slightly older than the average age of the published cases (i.e., 42 years) (Table 2). However, the presentation can be longer after the onset of the lesion due to its benign origin. According to other researchers, the age of onset of solitary FB is the sixth decade of life^(7)^, in contrast to the markedly earlier onset observed for the multiple hereditary form (i.e., the third decade of life). Reports of solitary FB in Korea revealed that the lesion can arise in patients between 1 and 36 years of age^(9^,^10)^.

Our case series was characterized by a female predominance (64%). However, according to the previous reported cases (Table 1) there is no sex preponderance in FB. Our case series was based on lesion exeresis and the female preponderance can be a bias related to the higher likelihood for women to consider lesion removal due to cosmesis.

Solitary FB is a rare condition typically arising as multiple lesions located in different areas, such as the scalp, forehead, face, neck, and upper trunk. Occurrence of this type of lesion on the eyelids is even more uncommon^(7)^. We observed only one case located on the eyelids, which indicated a frequency of 0.05 cases per year, considering our study period. Our solitary FB located on the eyelid is the third case reported thus far in the literature and was detected in a 72-year-old patient, while the previous lesions were reported in patients aged 37^(7)^ and 32 years^(1)^.

Solitary FB can exhibit various clinical features, without typical recognizable local or systemic symptoms^(7)^. Owing to its clinical similarities with other lesions, solitary FB lesions are generally diagnosed through histopathological examinations after excisional biopsy^(3)^. All our patients and also those from other reports were incorrectly diagnosed following simple ophthalmic examinations^(11)^.

In this case series, we only reported solitary FB cases. Solitary FB lesions include a spectrum of benign follicular neoplasms, with typical histologically features appea ring centered around a hair follicle that is occasionally dilated and contains keratin material surrounded by a moderately well-circumscribed thick mantle of fibrous tissue. The infundibular follicular epithelium extends out into this fibrous mantle forming epithelial strands or cords. The surrounding stroma consists of connective tissue with fibrillary collagen, fibroblasts, capillaries and, occasionally, mesenchymal mucin.

Differential diagnosis may be perifollicular fibroma, trichofolliculoma, trichoepithelioma, trichilemmoma, trichodiscoma, angiofibromas, and basaloid follicular hamartomas^(3^,^7)^. Trichodiscoma is a differential diagnosis, described as a well demarcated lesion with a predominant fibrous stroma^(12^. It is likely that FB and trichodiscoma are two different evolutionary stages of the same lesion and features of both neoplasms may occasionally be found in a single specimen^(13)^.

All our patients were treated using “shave” or complete excisional biopsy. Surgical removal is the mainstay of treatment for solitary FB (Table 2).

None of our patients experienced recurrence, although three patients did not present for a follow-up examination. In addition, patients should be screened for other cutaneous and extracutaneous features^(3)^.

In conclusion, solitary FB is a rare lesion, seldom affecting the lids. We reported 11 cases and the third case reported thus far in the literature affecting the lids. Diagnosis may be easily missed due to the nonspecific symptoms and clinical appearance. Therefore, it is necessary to perform excisional biopsy and histological examination for the recognition of this lesion.
